# Gene Expression Profiling and Biofunction Analysis of HepG2 Cells Targeted by Crocetin

**DOI:** 10.1155/2021/5512166

**Published:** 2021-04-01

**Authors:** Yi-Ling Wen, Yong Li, Guangcheng Zhu, Zhibing Zheng, Meng Shi, Si Qin

**Affiliations:** ^1^Lab of Food Function and Nutrigenomics, College of Food Science and Technology, Hunan Agricultural University, Changsha 410128, China; ^2^Department of Biochemical Science and Technology, Faculty of Agriculture, Kagoshima University, Korimoto 1-21-24, Kagoshima 890-0065, Japan

## Abstract

Crocetin is a carotenoid extracted from *Gardenia jasminoides*, one of the most popular traditional Chinese medicines, which has been used in the prevention and treatment of various diseases. The present study is aimed at clarifying the effect of crocetin on gene expression profiling of HepG2 cells by RNA-sequence assay and further investigating the molecular mechanism underlying the multiple biofunctions of crocetin based on bioinformatics analysis and molecular evidence. Among a total 23K differential genes identified, crocetin treatment upregulated the signals of 491 genes (2.14% of total gene probes) and downregulated the signals of 283 genes (1.24% of total gene probes) by ≥2-fold. The Gene Ontology analysis enriched these genes mainly on cell proliferation and apoptosis (BRD4 and DAXX); lipid formation (EHMT2); cell response to growth factor stimulation (CYP24A1 and GCNT2); and growth factor binding (ABCB1 and ABCG1), metabolism, and signal transduction processes. The KEGG pathway analysis revealed that crocetin has the potential to regulate transcriptional misregulation, ABC transporters, bile secretion, alcoholism, systemic lupus erythematosus (SLE), and other pathways, of which SLE was the most significantly disturbed pathway. The PPI network was constructed by using the STRING online protein interaction database and Cytoscape software, and 21 core proteins were obtained. RT-qPCR datasets serve as the solid evidence that verified the accuracy of transcriptome sequencing results with the same change trend. This study provides first-hand data for comprehensively understanding crocetin targeting on hepatic metabolism and its multiple biofunctions.

## 1. Introduction

Crocetin (*Gardenia jasminoides*) is a carotenoid originally extracted from a medicinal and edible resource and is one of the most popular traditional Chinese medicines. Crocetin has been used in the prevention and treatment of various diseases with health-promoting effects such as antioxidation, antitumor, anticardiovascular illness, anti-inflammation, hepatoprotection, and neuroprotection [1]. These therapeutic effects are achieved with different mechanisms. Accumulated data showed that crocetin could scavenge free radicals by inhibiting lipid peroxidation and increasing the activity of antioxidant enzymes including GST, GSH-Px, catalase, glutathione peroxidase (GPx), and superoxide dismutase (SOD) [2, 3]. Tumor invasion was suppressed by crocetin through reducing MMP expression and tumor proliferation by inducing cell cycle arrest, and enhancing apoptosis by activating caspase 3 [4, 5]. Cardiovascular disorders such as hypertension, thrombosis formation, and myocardial infraction (MI) could be potentially prevented or treated by crocetin. Moreover, crocetin effectively prevented inflammatory markers such as NF-*κ*B, TNF-*α*, IL-1*β*, IL-6, and IL-8 and inhibited iNOS/COX-2 activity and induction of NO/PGE2 [6–8]. The main mechanisms of the protective effects of crocetin in liver and nerve damage are mediated by the restoration of antioxidant enzyme activity and inhibition of the superoxide anion and/or free radical [9–11]. The nutritional value and therapeutic potential of crocetin have been gradually revealed, and it is therefore necessary to comprehensively and deeply analyze the molecular mechanism of the action of crocetin, and then fully reveal its functional activity.

Nutrigenomics is an emerging research field that studies the genomic changes caused by diet, covering the interaction of health, diet, and genomics. The human diet is composed of a complex mixture of substances and biological activity. It is functionally divided into three aspects: some can directly affect gene expression, some can modulate transcription factor activity after metabolism, while some can induce transcription after stimulating the signal transduction cascade [12]. Transcriptome refers to the sum of RNA transcribed by cells, tissues, or organisms at a certain developmental stage or under physiological conditions, mainly composed of messenger RNA (mRNA), noncoding RNA (ncRNA), and microRNA (miRNA). Transcriptomics is a discipline that systematically studies the global transcription map of the whole genome and reveals the network of molecular mechanisms of complex biological processes and trait regulation. A variety of techniques, including EST sequence construction and study, gene microarray, gene expression series analysis (SAGE), large-scale parallel sequencing (MPSS), and RNA-seq, have been developed to study the transcriptome. RNA-seq technology is established on the basis of EST technology and gene microarray. It is different from the gene expression microarray method in the sense that RNA-seq can not only detect transcripts corresponding to the existing genome sequence, but it can also find and quantify new transcripts, which have more advantages in the study of selective splicing events, new genes and transcripts, and fusion transcripts. RNA-seq has become the most popular method for transcriptome studies due to its low cost, its high throughput, and its applicability to species with no genomic background.

Recently, in the field of nutrition, there have been many studies on organisms, cells, or tissues under different nutritional states based on RNA-seq technology. Studies have reported that dietary composition can affect gene expression, thus affecting biological processes and pathways. Therefore, changing the dietary composition and proportion of farm animals could improve the nutritional value of meat products [13–15]. Suarez-Vega et al. [16] used the RNA-seq technique to study the MED-related mammary responses induced by CLA in breast milk, and compared the transcriptome data of mammary cells in ewes with the CLA-induced MFD group, the non-MED group, and the FO-MFD group, and identified the core genes involved in the downregulation of fatty acid synthesis in CLA-MFD and FO-MFD. Today, RNA-seq remains the preferred method of analysis for transcriptomes, and nutritionists are able to use transcriptome data to develop a framework for improving dietary composition and novel functional foods as a way to promote human health.

To study the effect of crocetin on the whole genome expression profile of human hepatocytes, RNA-seq technology was performed in HepG2 cells with or without crocetin treatment. The GO and KEGG pathway analysis has been applied to investigate the multiple biofunctions of crocetin. RT-qPCR was further used to verify the gene expression data. This study demonstrated the potential of using of crocetin as a functional ingredient applicable in medicine and food.

## 2. Materials and Methods

### 2.1. Materials, Cell Culture, and Cytotoxicity

Crocetin was provided by Tairui Biotechnology Co., Ltd. (Nanyang, Henan, China). LPS (*Escherichia coli* serotype 055:B5) was from Sigma-Aldrich (St. Louis, USA). Human hepatoblastoma HepG2 cells were purchased from ATCC (Rockville, USA) and cultured at 37°C in a 5% CO_2_ atmosphere in Dulbecco's modified Eagle's medium (DMEM; Gibco BRL, Gaithersburg, USA) containing 10% FBS. Fetal bovine serum (FBS) was from Biological Industries (Kibbutz Beit Haemek, Israel). 3-(4,5-Dimethylthiazol-2-yl)-2,5-diphenyl tetrazolium bromide (MTT) was purchased from Sigma-Aldrich (St. Louis, USA). MTT assay was used to check the cytotoxicity of crocetin. Briefly, HepG2 cells were seeded into a 96-well plate at a density of 10^4^ cells per well and preincubated at 37°C in 5% CO_2_ for 24 h. The cells were treated with a series of crocetin concentrations (0, 10 *μ*M, 20 *μ*M, and 40 *μ*M) for another 24 h, then MTT (final concentration 0.5 mg/ml) was added to the plate and incubated for 4 h in the dark. Acidic isopropanol (0.04–0.1 M HCl in isopropanol) was added to dissolve the formazan crystals, and the optical density (OD) was measured at 570 nm with a microplate reader (Thermo Fisher Scientific, Waltham, USA). Cell viability was measured by comparing the OD of crocetin-treated cells with that of untreated cells.

### 2.2. RNA Extraction and Construction of cDNA Library

HepG2 cells were precultured for 24 h and then were treated with 20 *μ*m crocetin dissolved in 0.1% DMSO for 8 h. Total RNA was extracted using the Isogen RNA kit (Nippon Gene Co., Tokyo, Japan) according to the manufacturer's protocol. The concentration and quality of RNA were assessed by a NanoDrop Spectrophotometer and an Agilent 2200 Bioanalyzer following the manufacturer's protocol. The cDNA of the purified mRNA was transformed and amplified by PCR technology, and the PCR products were purified by AMPure XP beads to complete the construction of the cDNA library. After the library construction was completed, the library was initially quantified (Qubit 2.0), then the insert fragment size of the library was detected using Agilent 2100, and the qualified cDNA libraries were sequenced on an Illumina HiSeq 2500 platform for high-throughput sequencing.

### 2.3. Bioinformatics Analysis

The R language package DESeq2 was used to screen differentially expressed genes. The significant difference standard for differentially expressed genes in this study is ∣log2 fold change | >0 and *p* value < 0.05, where the fold change value of the gene is the difference fold change, and the higher the value, the greater the difference in the expression of the gene between the two groups; the *p* value is the probability of hypothesis testing in the statistical model, and the smaller the *p* value, the greater the difference in expression between samples. DAVID is an online, web-based bioinformatics network analysis platform, which can realize fast, accurate, and comprehensive functional annotation analysis of large-scale gene or protein lists. GO and KEGG analysis methods are used to perform functional analysis on the differentially expressed genes, and to screen out differentially expressed genes enriched in pathways related to crocetin treatment.

### 2.4. Construction of Protein Interaction Networks

Functions and interactions between proteins were analyzed by the protein-protein interaction (PPI) system. The STRING database is the largest repository of its kind, retrieving millions of discoveries from full-text literature and is updated weekly. Using the Cytoscape software to construct the PPI network, setting the protein-protein interaction score greater than 0.7 has statistical significance. CytoHubba and MCODE were used to determine the core proteins in the complex protein interaction network, and clustering was used to construct functional modules.

### 2.5. Real-Time PCR

The genes with obvious differential expression were selected, respectively, and the corresponding sequence files were obtained, and the amplification primers of each gene were designed across exons using the Primer 5 software. The primer sequences were designed and applied for gene expression identification as shown in Table 1. Subsequently, these genes were verified by real-time fluorescent quantitative PCR (RT-qPCR). According to manufacturer's manual, the DyNAmo™ SYBR® Green 2-Step qRT-PCR Kit (Finnzymes Oy, Espoo, Finland) was used for reverse transcription and real-time PCR operations. Specifically, Oligo dT was used to reverse transcribe RNA (200 ng) into cDNA. M-MuLV RNase was applied at 37°C for 30 minutes, and then the reaction was terminated at 85°C for 5 minutes. PCR was determined according to the sequence of each primer with the Tm value. Each PCR contains 250 ng reverse transcript, 75 ng primer, and 10 *μ*l master mix. The thermal cycling conditions are maintained at 95°C for 15 min, and then at 94°C for 55 cycles of 30 s, with a Tm value of 30 s (melting temperature) and 30 sec at 72°C in a Rotor-Gene-3000AKAA (Corbett Research Pty., NSW, Australia).

### 2.6. Statistical Analysis

All the experimental data were expressed as mean ± SD. Statistical significance was analyzed by Student's *t*-test and ANOVA. A statistical probability of *p* < 0.05 was considered statistically significant.

## 3. Results

### 3.1. Gene Expression Profiling

**Figure 1:**
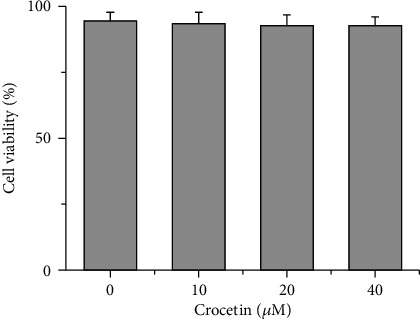
The cytotoxicity assay of HepG2 cells treated by crocetin (^∗^*p* < 0.05).

HepG2 cells were treated with diverse concentrations of crocetin, and the cytotoxicity was detected using a CCK-8 kit according the manufacturer's instructions. As shown in Figure 1, compared with the blank group, when the concentration of crocetin reached 40 *μ*M, the number of living cells had no significant change with the survival rate of cells above 90%, indicating that there was no toxicity to the cells under the concentration of crocetin in the range of 0-40 *μ*M.

**Figure 2:**
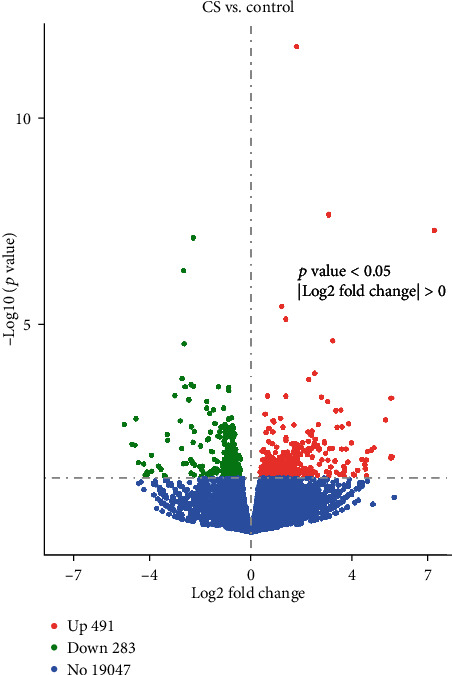
Volcano map of differentially expressed genes.

Among the total 45K genes, crocetin can affect the expressions of 19,821 genes among the 22,580 annotated genes in HepG2 cells; the disturbed fold of each gene is listed in Supplementary Table 1. As shown in Figure 2, the volcano map reflects the expression status of all the genes, in which green and red represent the downregulated and upregulated genes, respectively, and blue represents the genes with no differentially expressed genes. The total number of differential genes obtained from the experimental group and the control group was 774, among which 491 were upregulated genes and 283 were downregulated genes. The volcano map comparison showed that our method had sufficient coverage to detect differences in gene expression in the CS group relative to the control group.

Among the differentially expressed genes after crocetin treatment, the differentially expressed multiple of 6 genes was greater than or equal to 5, among which 5 genes were upregulated and 1 gene was downregulated. The differential expression multiple of 19 genes was greater than or equal to 4, among which 11 genes were upregulated and 8 genes were downregulated. There were 39 genes with differential expression ratios greater than or equal to 3, among which 28 genes were upregulated and 11 genes were downregulated. The differential expression ratio of 71 genes was greater than or equal to 2, among which 49 genes were upregulated and 22 genes were downregulated. In general, from the cells treated with crocetin, there were 135 genes (0.68%, 135 : 19821) with a differential expression ratio higher than or equal to 2 times.

### 3.2. Gene Ontology and KEGG Pathway Analysis

**Figure 3:**
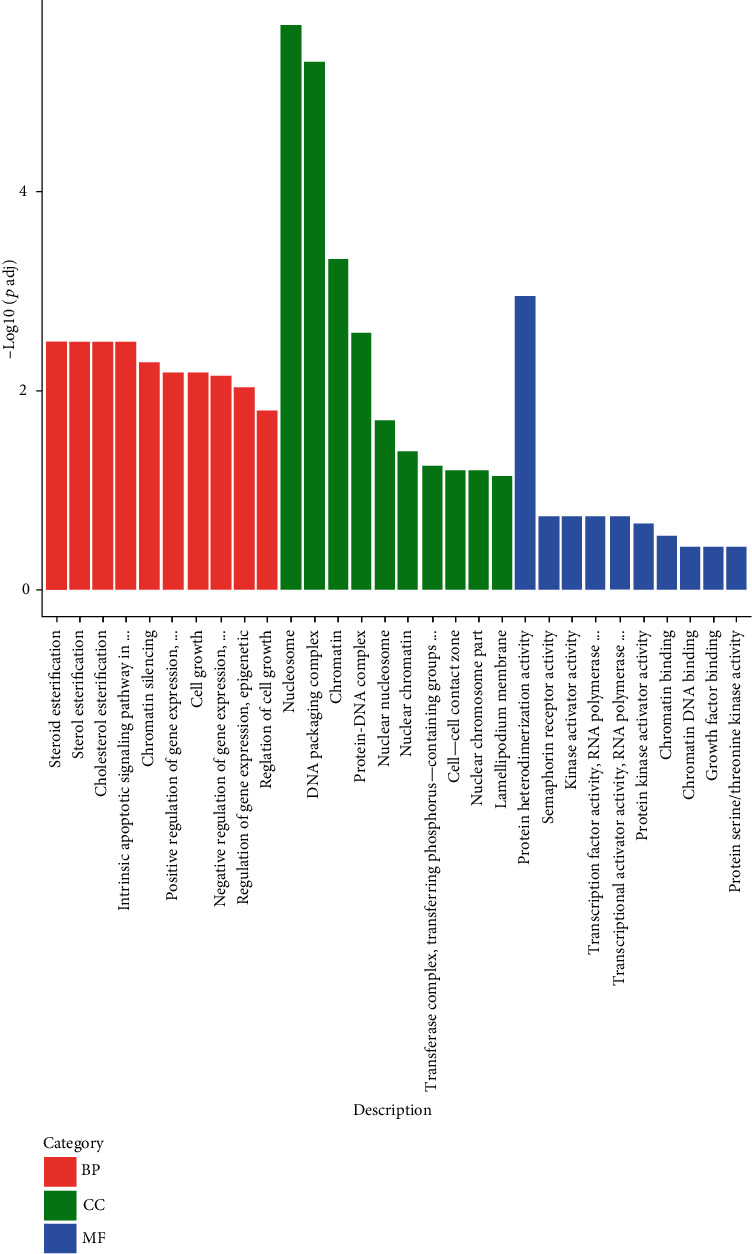
GO analysis of differentially expressed genes. GO analysis includes biological process (BP) (red), cell component (CC) (green), and molecular function (MF) (blue).

As shown in Figure 3, after CS treatment (control vs. CS), nucleosome and DNA packaging complex-related components were the most significantly disturbed in the cell component group (CC in green), and chromatin and protein-DNA complex-related processes were the most enriched differential genes; the numbers of differential genes were 44 and 22, respectively. From the molecular function group (MF in blue), the most significantly disturbed was the activity of the protein heterodimer, in which the number of differentially enriched genes was 42. The most significantly disturbed in the biological process group (BP in red) was sterol esterification, in which the numbers of differentially enriched genes related to cell growth and gene epigenetic regulation were 40 and 34, respectively.

**Figure 4:**
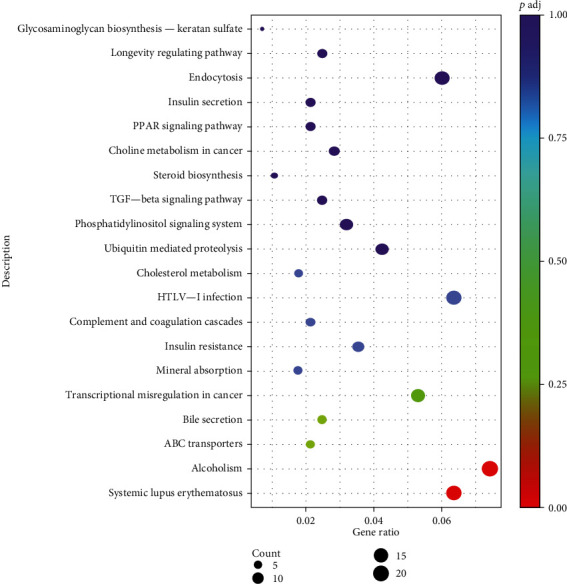
KEGG analysis of differentially expressed genes.

In order to further understand the functions and pathways of differential genes, the software Clusterprofiler was used to conduct KEGG pathway enrichment analysis of differential gene sets, and the top 20 pathways with the highest enrichment significance were selected for mapping. As shown in Figure 4, after CS treatment, 283 differential genes were compared with 251 KEGG pathways, which were related to transcriptional misregulation, ABC transporters, bile secretion, alcoholism, systemic lupus erythematosus, and other pathways. Among them, systemic lupus erythematosus (SLE) was the most significantly disturbed pathway, which is the first time it was implied that the crocetin has a potent and promising effect on this serious autoimmune disease.

### 3.3. PPI Network Analysis and Core Disturbed Gene Screening

The PPI network belongs to a scale-free network, which is not uniform. Among the PPI networks, most of the nodes have only one or two connections while a few nodes have lots of connections, ensuring that the system is fully connected. Nodes with a high number of connections, in this network, become hubs, which play an irreplaceable role in biological evolution and in maintaining the stability of the interaction network. These nodes normally have very key biological functions and participate in important life activities. The results show that the more interactions a protein interacts with, the more important is its role for the survival of the cell.

In order to understand the function of crocetin-induced differentially expressed genes and screen out Hub genes/proteins, the STRING database was used to analyze the interactions on the PPI network relationship. The list of differentially expressed genes was imported into the STRING database, and the data obtained from experiments, literatures, and high-throughput evidences were combined, and then the protein interaction data set encoded by the differentially expressed genes was downloaded. In the PPI network of differentially expressed genes treated with crocetin, 359 differentially expressed genes that encoded proteins were screened with relatively close interactions, including 359 protein nodes and 761 correlations.

**Figure 5:**
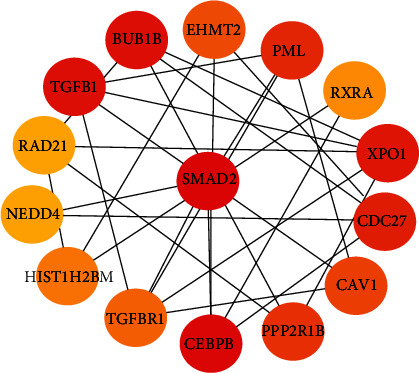
The 15 genes with the highest parameter values obtained by calculation are used as hub genes. The color change of the node indicates the increase or decrease of the parameter value.

However, it is difficult to visually identify the key nodes in the network due to the scale-free network nature of most biological data networks. CytoHubba plug-ins in the Cytoscape software were then used to calculate the Hub genes/proteins in the network diagram. According to the order of parameter and radiality from high to low as the standard, a total of 15 core proteins were screened, which includes the SMAD family variant 2 (SMAD2), CCAAT enhancer binding protein beta (CEBPB), conversion factor 1 (TGFB1), mitotic checkpoint serine/threonine protein kinase B (BUB1B), output 1 (XPO1), cell division cycle protein 27 (CDC27), early young granulocyte leukemia variant 1 (PML) transcription, protein phosphatase 2 support subunits X5 (PPP2R1B), cellar protein 1 (CAV1), autosomal histone lysine methylation transferase (EHMT2) 2, transforming growth factor beta receptor 1 (TGFbR1), histone (Hist1H2BM), retinol X receptor *α* (RXRA), adhesive protein complex (Rad21), and E3 ubiquitin protein ligase (Nedd4) (Figure 5). SMAD proteins encoded by their respective genes are signal transducers and transcriptional modulators that mediate multiple signaling pathways [17, 18]. This protein mediates the signal of the transforming growth factor- (TGF-) beta, and thus regulates multiple cellular processes, such as cell proliferation, apoptosis, and differentiation [19, 20]. SMAD proteins are recruited to the TGF-beta receptors through its interaction with the SMAD anchor for receptor activation (SARA) protein, and further phosphorylated by the TGF-beta receptors [21].

**Figure 6:**
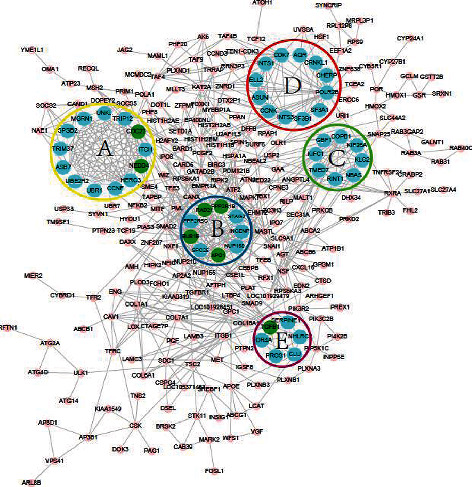
The five most important modules in the PPI network. According to the standard with a K-core greater than 6, the five important clusters obtained from formic acid in the PPI network are labeled A, B, C, D, and E, respectively, and are considered to be the topological center of the PPI network. The nodes marked in red represent the upregulated genes participating in the 5 subnetworks (none), and the nodes marked in green represent the downregulated genes.

For further understanding the important module of PPI, the Cytoscape MCODE software was used to screen out fine modules with K-core values greater than 6, and mark the above 15 hub genes in each module; we can see that the 15 hub genes were mostly in the five important modules selected as shown in Figure 6.

### 3.4. Regulation of the Expressions of Typical Genes by Crocetin Treatment

Drug metabolic enzymes are composed of phase I and phase II metabolic enzymes and phase III transporters, which play an important role in the metabolism, digestion, and detoxification of foreign substances and drugs. The biological functions of crocetin in the human body, such as anticancer, antioxidant, and anti-inflammatory, have been found, but its effect on liver cells, especially on hepatic drug metabolism enzymes and transporters, are still unclear. In the present study, genes with a differential expression ratio greater than or equal to 1.5 were selected as significantly different genes. Among the total 445 metabolic enzyme genes, 28 genes were disturbed by crocetin, with 20 genes upregulated and 8 genes downregulated. For instance, *GCNT2*, *ABCB1*, *ABCG1*, *ABCA2*, *CYP24A1*, *CYP27B1*, and *AHRR* are significantly upregulated genes by crocetin treatment, which implied that crocetin stimulated the expressions of hepatic metabolic enzymes.

In order to know the effect of crocetin on inflammation in the liver cells, several typical inflammatory differentially expressed genes were selected for analysis, and it was found that 199 genes in the total 455 inflammatory genes were significantly disturbed by crocetin treatment, with 158 upregulated genes and 41 downregulated genes. Among them, *GRP35*, *AGT*, *PDE5A*, *MMP28*, *IFIT2*, *TCF19*, *FGFBP1*, *SOCS5*, *TGFB1*, *PCGF2*, *FGFRL1*, *TNFRSF21*, and *VGF* are the most significantly downregulated genes by crocetin treatment, which revealed that crocetin is a potent and promising anti-inflammatory substance. Besides, glycolipid metabolism and other biofunction genes were also selected for analysis, and it was found that many glycolipid metabolism-related genes, 48 from the total of 445 genes, such as *EHMT2*, *AGT*, *RYR3*, *HBP1*, *GCNT2*, *BMT2*, *HSF1*, *NSF*, *TSC2*, *SP2*, *FA2H*, *CAD*, and *APOE*, were the most disturbed genes by crocetin treatment, which indicated that crocetin may exert its multiple biofunction by regulating glycolipid metabolism.

**Figure 7:**
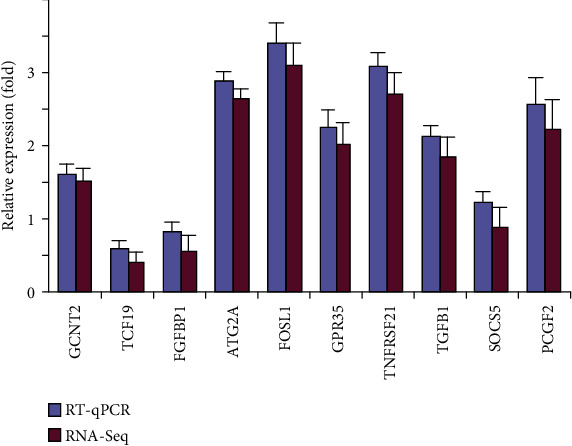
Comparison of differential gene expressions obtained by real-time PCR and RNA-seq sequencing results. Error bars show standard deviation.

Finally, to verify the accuracy of the transcriptome data, several typical genes are selected by RT-qPCR verification. As shown in Figure 7, the RT-qPCR data showed the same trends with the results of transcriptome, suggesting that RNA-sequencing is believable.

## 4. Discussion and Conclusion

The present study investigated the effect of crocetin on gene expression profiling of HepG2 cells by RNA-sequence assay and further analyzed the molecular mechanism underlying its multiple biofunction based on bioinformatics analysis and molecular evidence. The 774 differentially expressed genes in the transcription process were annotated by GO and KEGG analysis. In the three categories of the GO analysis catalog, the differentially expressed genes were mainly concentrated in nucleosomes, protein-DNA complexes, transcription factors, lipids, transferases, transcription factors, and other processes in the CC group. When treated with MF, different genes were mainly concentrated in the activities of signal transduction receptors, transcription factors, protein kinases, growth factors, transformation factors, etc. Differential genes were mainly concentrated in the MP group such as esterification, apoptosis, chromatin or gene silencing, and cell response to growth factor stimulation.

Through the PPI network analysis of the interaction between the coding proteins of different genes, the interaction between multiple differentially expressed genes was enriched. Further, 15 core proteins in the network were screened by the Cytoscape software, among which the differentially expressed genes corresponding to the core proteins of PPP2R1b, Rad21, Sub1b, XPO1, and TGFB1 were significantly downregulated. Among them, PPP2R1B is a tumor suppressor gene that encodes the *β* subtype of the serine/threonine-specific protein phosphatase 2A (PP2A-A*β*) A subunit, which is inactivated in cancer patients [22]. RAD21 is involved in the regulation of the normal cell cycle, DNA, DSB repair, and apoptosis pathways. In addition, the germline heterozygous or homozygous missense mutations of RAD21 are related to human genetic diseases [23]. XPO1 is an export receptor responsible for the nuclear-cytoplasmic transport of hundreds of proteins and various RNAs. XPO1 is often overexpressed and/or mutated in human cancers, and acts as a carcinogenic driver. Therefore, inhibiting XPO1-mediated nuclear export is a unique treatment strategy [24]. Subsequently, the PPI network of the modules was screened, and 5 important modules were obtained; most of these core proteins were in these 5 important modules.

To be noted, it was found that among the total drug metabolism enzymes (445 genes), 28 genes were significantly disturbed by crocetin treatment, with 20 genes upregulated and 8 genes downregulated, indicating that crocetin could positively influence the processes related to drug metabolism enzymes in human liver cells. Among them, *GCNT2* [25], *ABCB1* [26, 27], *ABCG1* [28, 29], *ABCA2* [30], *CYP24A1* [31, 32], *CYP27B1* [33], and *AHRR* [34] were the core genes of drug metabolism enzymes, which were worthy of further verification. Abnormal cellular proliferation and apoptosis are typical features of tumor cells, and genetic variations in the study pathway enrichment are mainly involved in transcription, intrinsic apoptotic signaling pathways, gene silencing, miRNA regulation gene silencing, and cell cycle regulation. Of these, 62 and 35 genes directly regulated the cell proliferation and apoptosis processes, respectively, and it is speculated that saffron acid could promote cell proliferation and apoptosis. In particular, *BRD4* [35, 36], *FAM83H* [37, 38], and *FOXK1* [39–42] are reported to be involved in the cell proliferation process of human pancreatic cancer and lung cancer, respectively, while *DAX* [43], *PML* [44], and *ULK1* [45] are reported to be involved in the cell apoptosis process of breast cancer, leukemia, and smooth muscle cells, respectively.

The above results indicate that crocetin is mostly positively associated with drug metabolism enzymes, transporters, inflammatory factors, and glucose and lipid metabolism, as well as cell proliferation and apoptosis processes in HepG2 cells. And the core genes were further detected by an RT-PCR experiment, which was consistent with the results of transcriptome sequencing, indicating that the results of transcriptome sequencing were real and reliable.

To conclude, our transcriptome data revealed gene expression profiles of crocetin in HepG2 cells for the first time. Signaling pathway analysis further demonstrated that systemic lupus erythematosus is involved in crocetin-induced expressions of most hepatic drug metabolizing enzyme genes. These results provide a comprehensive data for understanding the hepatic metabolism, the bioactive role, and the molecular mechanisms of crocetin.

## Figures and Tables

**Table 1:** The primers used for real-time PCR.

Oligonucleotide	Sequence (5′-3′)
FGFBP1-F	CTTCACAGCAAAGTGGTCTCA
FGFBP1-R	GACACAGGAAAATTCATGGTCCA
GCNT2-F	TGTTCCTGGCTCTATGCCAAA
GCNT2-R	TTAGCAAACAGGCTTGGTGAAT
GPR35-F	GGGAGGACCGTCTGCACAAA
GPR35-R	CCCAGGTGGCTGAATCTGGTG
TNFRSF21-F	GCCAGTGAGAGGGAGGTTGC
TNFRSF21-R	TCCAGCTGGGTGGTGTCTTC
TGFB1-F	GCGTCTGCTGAGGCTCAAGT
TGFB1-R	GCCGGTAGTGAACCCGTTGAT
TCF19-F	GGGGCGGTGATCTCTACAC
TCF19-R	GGGAGTCGGACATTATTGACCA
PCGF2-F	GCGAGGTCTTGGAGCAGGAG
PCGF2-R	GGCGATGTCCATGAGGGTGT
GAPDH-F	CATGGCACCGTCAAGGCTGA
GAPDH-R	ACGTACTCAGCGCCAGCATC
ATG2A-F	CTCGCCTCCTCCCAGATCAA
ATG2A-R	GGGCATCCTGGTCCACATTG
FOSL1-F	CAGGCGGAGACTGACAAACTG
FOSL1-R	TCCTTCCGGGATTTTGCAGAT
SOCSS-F	GTGCCACAGAAATCCCTCAAA
SOCSS-R	TCTCTTCGTGCAAGTCTTGTTC

## Data Availability

The transcriptome data used to support the findings of this study are included within the supplementary information file.

## Supplementary Materials

Supplementary Materials Supplementary description: raw data of the transcriptome assay in HepG2 cells.
