# Supplementary material for: Gene Expression Profiling and Biofunction Analysis of HepG2 Cells Targeted by Crocetin
Source: Mediators Inflamm. 2021 Apr 1;2021:5512166. doi: 10.1155/2021/5512166 (PMC8035019; doi:10.1155/2021/5512166)
Supplement: Supplementary Materials — Supplementary description: raw data of the transcriptome assay in HepG2 cells. [file 5512166.f1.docx]

Supplementary description.

raw data of the transcriptome assay in HepG2 cells
